# Adherence to aerobic training combined with high protein intake is associated with low blood pressure in Italian older adults: a cross-sectional study

**DOI:** 10.1007/s40520-023-02549-x

**Published:** 2023-09-08

**Authors:** Hélio José Coelho-Júnior, Riccardo Calvani, Anna Picca, Matteo Tosato, Giulia Savera, Francesco Landi, Emanuele Marzetti

**Affiliations:** 1 Department of Geriatrics, Orthopedics and Rheumatology, Università Cattolica del Sacro Cuore, 00168 Rome, Italy; 2 Fondazione Policlinico Universitario “A. Gemelli” IRCCS, 00168 Rome, Italy; 3 Department of Medicine and Surgery, LUM University, 70100 Casamassima, Italy

**Keywords:** Cardiovascular disease, Physical exercise, Swimming, Running, Physical activity, Elderly

## Abstract

**Background:**

Lifestyle habits have a key role in cardiometabolic health. The effects of combined aerobic training (AT) and high protein intake (HPI) on cardiometabolic parameters in older adults are not well established.

**Aims:**

To investigate the association of AT and HPI with blood pressure (BP), blood glucose, and total blood cholesterol levels in a sample of Italian older adults enrolled in the Longevity Check-up 7 + (Lookup 7 +) study.

**Methods:**

Lookup 7 + is an ongoing project started in June 2015 and conducted in unconventional settings (e.g., exhibitions, malls, health promotion campaigns) across Italy with the aim of fostering adoption of healthy lifestyles in the general population. For the present investigation, analyses were conducted in participants 65 + years and with body mass index values ≥ 18.5 kg/m^2^ (n = 3219). Systolic (SBP) and diastolic BP (DBP), blood glucose, and total blood cholesterol were measured. Protein intake was estimated using a 12-item food frequency questionnaire. HPI was operationalized as a daily protein intake ≥ 0.8 g/kg of body weight. AT was operationalized as the practice of running and/or swimming for 60 + minutes at least twice weekly during the previous year.

**Results:**

The mean age of the 3219 participants was 72.7 ± 5.7 years, and 55.2% were women. Adherence to AT combined with a HPI was negatively and independently associated with SPB (β: − 4.976; 95% confidence interval: − 9.8 to − 0.08). No other significant associations were observed.

**Discussion and conclusions:**

Our results indicate that AT combined with HPI was negatively associated with SBP in a large and relatively unselected sample of Italian older adults living in the community. These findings need confirmation by ad hoc designed studies.

## Background

Cardiovascular disease (CVD) is the leading cause of death around the world, killing almost 20 million people every year [1]. Older adults are highly affected by CVD, with ~ 70% of those 65 years and older showing some cardiometabolic risk factors [2, 3]. CVD burden in older adults is concerning due to its association with hospitalizations, healthcare costs, and mortality [2, 3]. The development of CVD in advanced age occurs as a consequence of age-related dysregulations of cardiometabolic homeostasis [4], encompassing high blood pressure (BP), abnormal glucose metabolism, and hyperlipidemia.

Physical activity and nutrition are two major modifiable lifestyle factors that may be harnessed for the prevention and treatment of cardiometabolic diseases [5–9]. Numerous studies have reported that aerobic training (AT), land- or water-based exercises in which muscle contractions are sustained for long periods [10], ameliorates cardiometabolic risk factors [11–14]. This evidence led experts in the field to recommend exercise training as part of non-pharmacological treatment for cardiometabolic diseases [5, 6, 9]. However, the effectiveness of AT is not supported by all studies [11–14], and more investigations are required.

The adherence to some dietary patterns also brings health benefits to the cardiovascular system. Besides carbohydrates and fats, dietary protein affects cardiometabolic parameters. However, the effects of protein intake vary according to their amino acid composition [15–17] and the simultaneous consumption of other macronutrients [18]. Indeed, conflicting results have been reported, with studies showing positive, negative, and null relationships between protein consumption and cardiometabolic parameters [19–30].

A few investigations tested the effects of exercise training combined with a high protein intake (HPI) on cardiometabolic risk factors. Amamou et al. [31] reported that a 16-week dynamic resistance training program combined with HPI did not produce additional reductions in cardiometabolic risk factors than HPI alone. However, the study involved a small sample size and did not include an exercise control group.

Randomized clinical trials are expensive and time-consuming, and new interventions are tested when evidence supports the investment and allows the design of the best approach. Currently, there is not sufficient information to support the conduction of a randomized clinical trial to test the effects of exercise combined with HPI on cardiometabolic health. On the other hand, observational studies allow examination of numerous covariates and mixed samples of people with different conditions. Such an approach is an excellent method to collect information for designing clinical trials.

## Aims

The present study was conducted to investigate the association between AT and HPI with BP, blood glucose levels, and total blood cholesterol concentrations in a large sample of Italian older adults living in the community.

## Methods

Data of the present investigation were gathered from the Longevity Check-up 7 + (Lookup 7 +) project database. Sampling characteristics, procedures, and other results have been published elsewhere [32–39]. Recruitment was conducted among people visiting public spaces (e.g., exhibitions, shopping centers) and those adhering to prevention campaigns promoted by our institution [34]. The Lookup 7 + protocol was approved by the Ethics Committee of the Università Cattolica del Sacro Cuore (protocol #: A.1220/CE/2011) and each participant provided written informed consent prior to enrolment. The manuscript was prepared in compliance with the STrengthening the Reporting of Observational studies in Epidemiology (STROBE) guidelines for observational studies [40].

### Participants

From 1 June 2015 to 31 October 2021, 13,515 community-dwelling adults aged 18 + years were enrolled. Exclusion criteria were inability or unwillingness to provide written informed consent, self-reported pregnancy, and inability to perform the physical function tests. For the present investigation, analyses were conducted in participants aged 65 + years, with body mass index (BMI) values ≥ 18.5 kg/m^2^, and no missing data for the variables of interest, totaling 3219 participants (10,296 excluded).

All participants received a structured questionnaire to collect information on personal characteristics and lifestyle habits, followed by measurement of anthropometric parameters. The BMI was calculated as the ratio between body weight (BW, kg) and the square of height (m^2^). An oscillometric monitor was used to measure BP (Omron M6 electronic sphygmomanometer, Omron, Kyoto, Japan) [41]. Blood concentrations of glucose and total cholesterol were measured from capillary blood samples using disposable electrode strips based on a reflectometric system with a portable device (MultiCare-In, Biomedical Systems International Srl, Florence, Italy) [42]. Participants were asked if they were fasting for at least 8 h. A food frequency questionnaire (FFQ) was used to collect information on how often participants consumed a standardized portion size of a list of 12 foods (meat, meat derivatives, fish, eggs, milk, cheese, yogurt, pasta, bread, rice, vegetables, cereals) on a weekly basis [42]. The mean daily intake of protein was calculated by multiplying the consumption frequency of a food item by the protein content of its standard portion [42], then dividing by seven (the days of a week), followed by the sum of all applicable items. HPI was defined as a protein consumption equal or greater than the recommended dietary allowance (RDA) (≥ 0.8 g/kg of BW per day). Physically inactive (PI) and exercise groups were operationalized based on self-report. Those who did not practice ≥ 60 min at least twice weekly during the previous year were defined PI. Engagement in AT was operationalized as involvement in running and/or swimming activities [38]. The interview did not include questions about exercise frequency, volume, intensity, or length of engagement in AT. Smoking status was categorized as active smoker (has smoked 100 + cigarettes in lifetime and currently smokes cigarettes) and no current smoker.

### Statistical analysis

The normal distribution of variables was ascertained via the Shapiro–Wilk test. Continuous variables are expressed as the mean ± standard deviation (SD) or absolute numbers, percentages. Regression analysis was conducted to test the association between AT with or without HPI and cardiometabolic risk factors. PI was used as the reference group. The final model was adjusted for age, sex, BMI, energy intake, sodium, potassium, calcium, magnesium, active smoking, fasting state (blood glucose), and antihypertensive (for systolic and diastolic BP), cholesterol-lowering (for total blood cholesterol), and antidiabetic (for blood glucose) drugs. Significance was set at 5% (p < 0.05) for all tests. Significant results on regression analyses were expected to include a 95% confidence interval (CI 95%) that did not encompass the value of 1. All analyses were performed using the SPSS software (version 23.0, SPSS Inc., Chicago, IL, USA).

## Results

Three-thousand two-hundred nineteen older adults of the Lookup 7 + database met the eligibility criteria to be included in the present study. The main characteristics of study participants according to adherence to AT and protein intake groups are shown in Table 1. Personal and anthropometric characteristics of participants 65 + years who were not included in the analyses (n = 206) did not significantly differ from those analyzed. Participants engaged in AT were younger than those in the PI group, whereas individuals with HPI were older than those who only practiced AT. HPI groups had greater absolute and adjusted protein consumption than PI and AT. Similar results were observed across groups for energy, calcium, magnesium, sodium, and potassium intake. Lower diastolic PB values and higher cholesterol levels were found in participants in the HPI group relative to PI. Significant differences in the frequency of women and participants on antihypertensive and cholesterol-lowering treatments were observed across groups.

**Table 1:** Main characteristics of study participants according to physical activity and protein intake categories (n = 3219)

	PI (n = 2403)	AT (n = 433)	HPI (n = 335)	HPI + AT (n = 48)
Age, years	72.8 ± 5.7	71.6 ± 5.2^a^	73.6 ± 6.1^b^	70.9 ± 4.5^a^
Weight, kg	73.7 ± 12.8	73.3 ± 11.4	58.0 ± 9.0^ab^	58.4 ± 10.6^ab^
Height, m	1.64 ± 0.9	1.67 ± 0.9^a^	1.57 ± 0.7^ab^	1.59 ± 0.9^ab^
BMI, kg/m^2^	27.3 ± 4.1	26.1 ± 3.4	23.4 ± 3.1	22.9 ± 3.9
Sex, female	1301, 54.1	162, 37.4	280, 83.6	35, 73.0*
Energy intake, kcal	506.8 ± 128.3	449.7 ± 133.3	722.4 ± 122.1^ab^	710.2 ± 140.8^ab^
Protein, g	36.3 ± 8.0	35.9 ± 9.5	54.4 ± 8.7^ab^	53.2 ± 9.5^ab^
Protein, g/kg	0.50 ± 0.11	0.49 ± 0.12	0.94 ± 0.15^ab^	0.91 ± 0.11^ab^
Calcium, mg	256.8 ± 125.4	243.9 ± 118.2	404.7 ± 125.1^ab^	405.3 ± 102.6^ab^
Magnesium, mg	41.0 ± 11.4	39.3 ± 12.0	56.6 ± 11.4^ab^	55.4 ± 10.2^ab^
Sodium, mg	573.2 ± 245.9	575.1 ± 248.9	874.0 ± 325.6^ab^	818.8 ± 329.1^ab^
Potassium, mg	462.3 ± 125.2	460.8 ± 140.4	671.9 ± 146.5^ab^	655.7 ± 160.1^ab^
Systolic blood pressure, mmHg	130.8 ± 15.8	130.2 ± 15.5	130.6 ± 16.9	124.2 ± 17.1^a^
Diastolic blood pressure, mmHg	76.2 ± 9.6	76.1 ± 9.3	74.4 ± 9.7a	72.9 ± 8.9
Total blood cholesterol, mg/dL	198.1 ± 32.8	199.3 ± 31.3	206.5 ± 35.0^a^	308.8 ± 37.0
Blood glucose, mg/dL	107.6 ± 25.9	104.4 ± 21.2	104.3 ± 22.5	111.7 ± 46.8
Smoking, active	321, 13.3	59, 13.6	36, 10.7	6, 12.5
Physical activity, yes	1815, 75.5	332, 76.6	260, 77.6	35, 73.0
Antihypertensive drug(s), yes	1004, 41.7	233, 53.8	186, 55.5	26, 54.2*
Cholesterol-lowering drug(s), yes	1536, 64.0	313, 72.2	234, 70.0	33, 68.7*
Antidiabetic drug(s), yes	2153, 89.6	403, 93.0	312, 93.1	42, 87.5

^a^p < 0.05 vs. PI; ^b^p < 0.05 vs. AT; *p < 0.05 for the chi-square test

*AT* aerobic training, *BMI* body mass index, *HPI* high protein intake, *PI* physically inactive

Results of the linear regression for the association of adherence to AT with or without HPI and BP, blood glucose, and total blood cholesterol are shown in Tables 2, 3, 4, respectively. In the fully adjusted model, the practice of AT combined with a HPI was negatively associated with systolic BP values. No other significant associations were found.

**Table 2:** Association of aerobic training with or without high protein intake and blood pressure

	Univariate β (95% CI)	Adjusted β (95% CI)		Univariate β (95% CI)	Adjusted β (95% CI)		Univariate β (95% CI)	Adjusted β (95% CI)
SPB, mmHg								
PI	1.0 (Reference)	1.0 (Reference)	PI	1.0 (Reference)	1.0 (Reference)	PI	1.0 (Reference)	1.0 (Reference)
AT	– 0.602 (– 2.3, 1.1)	– 0.145 (– 1.9, 1.6)	HPI	– 0.168 (– 2.0, 1.7)	– 1.334 (– 1.2, 3.8)	HPI + AT	**– 6.608 (– 11.1, – 2.0)**	**– 4.976 (– 9.8, – 0.08)**
DBP, mmHg								
PI	1.0 (Reference)	1.0 (Reference)	PI	1.0 (Reference)	1.0 (Reference)	PI	1.0 (Reference)	1.0 (Reference)
AT	– 0.088 (– 1.1, 0.9)	0.073 (– 1.0, 1.1)	HPI	**– 1.856 (– 2.9, – 0.7)**	– 0.808 (– 2.3, 0.73)	HPI + AT	**– 3.348 (– 6.1, – 0.5)**	– 2.385 (– 5.3, 0.5)

Models are adjusted for age, sex, body mass index, energy intake, sodium, potassium, calcium, magnesium, walking activity, smoking status, and antihypertensive therapy. Bold denotes statistical significance based on p value and 95% confidence interval

*AT* aerobic training, *CI* confidence interval, *DBP* diastolic blood pressure, *HPI* high protein intake, *PI* physically inactive, *SBP* systolic blood pressure

**Table 3:** Association of aerobic training with or without high protein intake and blood glucose

	Univariate β (95% CI)	Adjusted β (95% CI)		Univariate β (95% CI)	Adjusted β (95% CI)		Univariate β (95% CI)	Adjusted β (95% CI)
Glucose, mg/dL								
PI	1.0 (Reference)	1.0 (Reference)	PI	1.0 (Reference)	1.0 (Reference)	PI	1.0 (Reference)	1.0 (Reference)
AT	**– 3.222 (– 6.0, – 0.4)**	– 2.034 (– 4.7, 0.6)	HPI	**– 3.302 (– 6.2, – 0.3)**	0.08 (– 3.78, 3.95)	HPI + AT	4.0 (– 3.5, 11.6)	6.3 (– 1.4, 14.0)

Models are adjusted for age, sex, body mass index, energy intake, sodium, potassium, calcium, magnesium, walking activity, smoking status, and antidiabetic therapy. Bold denotes statistical significance based on p value and 95% confidence interval

*AT* aerobic training, *CI* confidence interval, *HPI* high protein intake, *PI* physically inactive

**Table 4:** Association of aerobic training with or without high protein intake and total blood cholesterol

	Univariate β (95% CI)	Adjusted β (95% CI)		Univariate β (95% CI)	Adjusted β (95% CI)		Univariate β (95% CI)	Adjusted β (95% CI)
Cholesterol, mg/dL								
PI	1.0 (Reference)	1.0 (Reference)	PI	1.0 (Reference)	1.0 (Reference)	PI	1.0 (Reference)	1.0 (Reference)
AT	1.188 (– 2.5, 4.8)	0.447 (– 3.1, 4.0)	HPI	**8.400 (4.5, 12.2)**	4.58 (– 0.66, 9.82)	HPI + AT	**10.6 (1.2, 20.1)**	7.4 (– 2.4, 17.3)

Models are adjusted for age, sex, body mass index, energy intake, sodium, potassium, calcium, magnesium, walking activity, smoking status, and cholesterol-lowering therapy. Bold denotes statistical significance based on p value and 95% confidence interval

*AT* aerobic training, *CI* confidence interval, *HPI* high protein intake, *PI* physically inactive

## Discussion

Findings of the present study indicate that adherence to AT alone was not associated with cardiometabolic benefits in a sample of community-dwelling Italian older adults enrolled in Lookup 7 + . However, lower systolic BP values were observed in those who engaged AT and had a HPI relative to the PI group. No significant associations were observed between AT + HPI and blood glucose or total blood cholesterol levels.

To the best of our knowledge, this is the first study that investigated the association between AT combined with HPI and cardiometabolic risk factors. A few investigations have been conducted on the subject. Amamou et al. [31] did not observe additional benefits on glucose, triglycerides, total cholesterol, or BP levels in older adults when a high-protein diet was combined with a 16 week dynamic resistance training program. In a study testing the acute effects of protein intake on cardiovascular responses to exercise tests, Bergia et al. [43] found that oral supplementation with 30 g of protein did not affect BP or nitric oxide bioavailability. Similar results were found in a randomized crossover trial testing the acute effects of a protein-rich drink in exercising young men [44].

The chronic effects of walking/running [45] and aquatic activities [46] on cardiometabolic parameters have been widely tested and meta-analyzed [11–14, 47].﻿ Overall, results from a rigorous pooled analysis indicate that land- and water-based aerobic exercises may significantly reduce BP in older adults with different characteristics [48, 49]. Beneficial effects have also been observed on blood glucose and cholesterol levels [11–14, 50].

Differences between the results of the present study and the literature might be explained by exercise modalities and participant characteristics. Amelioration of cardiometabolic risk factors in response to exercise training is not easily achieved, and specific combinations of exercise variables might be required [51, 52]. Indeed, the effects of AT are more likely to be observed in women who perform moderate-to-high intensity programs at least 3 days a week for no less than 12 weeks [11]. The exercise volume (i.e., amount of activity) may also impact cardiometabolic parameters, with high-volume AT yielding larger reductions in blood cholesterol and glucose levels than moderate-volume AT [53] or high-intensity interval training [54]. Significant reductions in BP after swimming activities are observed when this exercise encompasses an endurance component, intermittent exercises, or specific swimming techniques (e.g., breaststroke kick) and is performed by hypertensive people [46]. Benefits of swimming on blood cholesterol are only observed for endurance-based activities and in young and middle-aged adults or those with dyslipidemia [50].

Based on these observations, it may be hypothesized that AT sessions of Lookup 7 + participants were not performed with sufficient frequency, intensity, or volume to elicit substantial cardiometabolic benefits. This view is supported by the lack of significant associations in the unadjusted analysis. However, exercise variables were not controlled for in the present study, which impedes from drawing solid conclusions. An increased access to adapted public spaces, the availability of inexpensive and easy-to-use equipment [55, 56], and the popularity of social media influencers [51] induce a growing number of individuals to exercise autonomously. However, supervised exercise may be necessary to elicit favorable adaptations [52].

The association between protein intake and cardiometabolic parameters has been widely explored. In relation to BP, investigations have produced conflicting results, with studies reporting positive, negative, or null relationships [19–30]. Protein intake was not associated with changes in BP or the incidence of hypertension in Dutch adults [25, 26]. Liu et al. [28] expanded these findings by reporting no associations of protein intake with the prevalence of hypertension in poorly nourished rural Chinese adults. Increased protein intake also failed to ameliorate hemodynamic parameters in randomized clinical trials [57]. In contrast, large cohort studies reported a negative association between protein intake and BP levels [19, 20, 23, 29, 57, 58].

Studies have also reported no associations between protein intake and blood lipid profile [22, 57]. Kohansal et al. [22] found no associations between protein intake and blood cholesterol levels in Iranian adults. Similar findings were reported by Tayyem et al. [57] in Jordanian adults. These divergent results might be due to protein consumption levels and amino acid composition.

Observation-based models have been proposed to explain the possible effects of protein intake on BP levels [59, 60]. Accordingly, individuals with HPI are expected to display low BP values, whereas those with moderate-to-low consumption might present normal or even high BP levels [59, 60]. Noticeably, participants of the present study reported a limited protein ingestion as shown by the small number of individuals in the HPI group and the low median protein intake in the whole sample (0.55 g/kg of BW). These observations suggest that, in our study population, the intake of protein might not be sufficient to lower BP.

The amino acid composition of dietary protein may have an influence on blood glucose and cholesterol levels. Hepatocytes incubated with homocysteine show increased cholesterol synthesis [61], and homocysteine intake is associated with greater cholesterol concentrations in the blood and liver [61, 62]. Rats fed a cystine-enriched diet displayed elevated liver, plasma, and serum cholesterol levels than controls [63, 64]. In contrast, taurine supplementation reduces total and low-density lipoprotein-cholesterol in rats on a high-cholesterol diet [65] and humans [66]. Similarly, leucine reduces systemic and liver cholesterol concentration in rats [67]. Hence, it is possible that participants of the present study might consume dietary protein whose amino acid composition was inadequate to ameliorate cardiometabolic parameters.

A question that remains is why the combination of AT and HPI was associated with low systolic BP, while either of them alone was not. A possible explanation is that the association of AT and HPI might improve endothelium-dependent vasodilation and increase blood flow and nutrient supply to muscle [68]. The greater metabolic demands of exercising muscles stimulate vasodilation and increase blood flow through a cascade of events that aims at matching the nutrient and oxygen supply to maintain physical work [69]. A repeated exposure to such an adaptation may eventually induce structural and functional alterations of the vascular bed that attenuate or even prevent the age-related decline in endothelium-dependent vasodilation [69, 70]. These changes in vascular structure and function might favor the vasodilating effects of dietary amino acids, such as l-arginine [71, 72]. Exercise may also reduce the concentration of l-arginine competitors for nitric oxide production [73, 74]. An alternative explanation is that amino acid ingestion, mainly branched-chain amino acids [75] combined with adequate AT designs [76, 77], may promote muscle hypertrophy. Indeed, community-dwelling older adults with low muscle mass frequently show elevated BP values and a high prevalence of hypertension [78, 79].

Although reporting novel findings, the present study is not free of limitations. First, our sample was exclusively composed of relatively young community-dwelling Caucasian older adults with well-controlled BP levels, and extrapolations to individuals of other ethnicities or with different age or BP values should be made with caution. In particular, ethnicity and hypertensive status may influence BP responses to exercise [49]. Second, studies have indicated that combined exercise protocols (aerobic plus resistance) produce greater improvements in cardiometabolic profile than individual exercise modalities [12, 13, 80]. Third, the FFQ used in the present study only included the weekly consumption of 12 food groups. This might explain the low protein intake observed in our study participants. Fourth, a meta-analysis found that high-protein diets did not affect fasting blood glucose but decreased HbA1C levels [81]. Hence, future studies should include more complex metabolic analyses to provide a deeper understanding of adaptations induced by dietary and exercise regimens. Fifth, participants were evaluated while they were attending events, in which they had to stand up and walk. Thus, we cannot exclude that the evaluation setting could have influenced BP values. Sixth, the presence of orthostatic hypotension [82] was not explored, and its impact on study results might not be ruled out. Seventh, participants with cardiovascular or cerebrovascular disease were not excluded. Eighth, data on physical activity habits were collected through self-report using simple questions embedded in the Lookup 7 + lifestyle questionnaire [38], instead of established tools for evaluation of physical activity in older adults (e.g., PASE, CHAMP, PAQE). Exercise variables (e.g., frequency, volume, intensity, length of engagement) were not collected or controlled for. Furthermore, subjective measures of physical activity may lead to over- or underestimation owing to inaccurate recall, misinterpretation, and response bias [83]. Limitations concerning physical activity and exercise assessment, albeit not negligible, are intrinsic to the design of Lookup 7 + and the unconventional settings where the survey is conducted. The collection of detailed information on lifestyle habits would substantially increase the duration of the assessments, which may deter many candidates from participating. Finally, the cross-sectional design of the study does not allow inference to be drawn on the time course of changes in the variables considered and on cause-effect relationships.

## Conclusion

Findings of the present study indicate that AT combined with HPI is negatively associated with systolic BP in a large and relatively unselected sample of community-dwelling Italian older adults. These results, albeit promising, need to be confirmed by ad hoc designed studies using more established tools for assessing physical activity habits in older adults. Further research is also warranted to explore the effects of interventions involving AT with HPI in older adults with a high cardiometabolic risk profile.

## Data Availability

The datasets generated and analyzed during the current study are available from the corresponding author on reasonable request.
